# Mechanistic Role of Astrocytic Rac1 Protein in Alzheimer's Disease

**DOI:** 10.14336/AD.2025.0502

**Published:** 2026-06-14

**Authors:** Fengwen Jiang, Niya Wang, Qiang Meng

**Affiliations:** ^1^Department of Neurology, The Affiliated Hospital of Kunming University of Science and Technology, The First People's Hospital of Yunnan Province, Kunming 650500, China.; ^2^School of Medicine, Yunnan University, Kunming, Yunnan, China.

**Keywords:** Rac1 protein, Alzheimer's disease, pathogenesis, TLR4/Rac1/NLRP3 signaling pathway, astrocytes

## Abstract

The prevalence of Alzheimer's disease (AD) has been increasing worldwide due to the aging population, placing a substantial burden on both society and families. To date, the underlying pathogenesis of AD has not been comprehensively elucidated, and advancements in drug development for this disease have been relatively slow. Astrocytes are crucial for maintaining the homeostasis of the brain microenvironment. Astrocyte dysfunction has been closely linked to AD onset and progression. The Rac1 protein, which belongs to the Rho GTPase family, exhibits hyperactivation in the astrocytes of AD model mice. Nevertheless, the exact role of Rac1 in the pathogenesis of AD remains ambiguous. The TLR4/Rac1/NLRP3 signaling pathway is involved in diverse cellular activities and inflammatory responses and plays a significant role in the AD neuroinflammatory process. This review explores the mechanism of action of Rac1 in astrocytes in the context of AD and how the TLR4/Rac1/NLRP3 pathway influences the pathological process, offering novel theoretical foundations and potential therapeutic targets for preventing and treating this disease.

## 1. Introduction

Alzheimer's disease (AD) is a common neurodegenerative disorder [1] characterized by short-term memory loss, progressive cognitive impairment, behavioral and psychological aberrations, and a decline in the capacity for daily living activities [2,3]. The principal pathological hallmarks of AD are linked to extracellular amyloid-β (Aβ) aggregation and intracellular Tau protein phosphorylation in neurons [4-6]. The prevalence of AD is increasing worldwide due to the aging population; currently, more than 50 million individuals have dementia globally [7]. Projections suggest that by 2050, the number of AD patients will exceed 100 million [8]. Nevertheless, the etiological factors of AD remain undefined.

Astrocytes, the largest glial cell population in the brain, exhibit functional diversity and high heterogeneity, playing indispensable roles in maintaining the structural and functional integrity of the nervous system [9]. These cells actively support neuronal energy metabolism through the glycolysis-lactate shuttle, which supplies high-energy metabolites (e.g., lactate) and metabolic substrates to neurons [10]. Additionally, astrocytes regulate cerebral blood flow (CBF) and blood-brain barrier (BBB) integrity [11], modulate extracellular ion homeostasis and neurotransmitter/fluid balance [12-14], and fine-tune synaptic plasticity [15,16]. As an integral part of the BBB, astrocytes play a significant role in controlling the outward turnover of Aβ in the brain as well as the transportation of peripheral Aβ into the brain [17,18]. During astrocyte dysfunction, the outward turnover of Aβ in the brain is reduced, resulting in Aβ accumulation and plaque formation, which accelerates the AD pathological process. Moreover, astrocytes are closely related to neuroinflammation. Under pathological conditions, astrocytes become activated and generate and release various inflammatory factors, including tumor necrosis factor-α (TNF-α), interleukin-1β (IL-1β), interleukin-6 (IL-6), and C-C motif chemokine ligand 2 (CCL2) [19]. These factors can further exacerbate the inflammatory response. How to exert the phagocytic function of astrocytes without triggering the release of inflammatory factors remains a challenge for research.

Rac1, a member of the Ras homologous (Rho) GTPase family of small guanosine triphosphatases (G-proteins), exists in two conformational states: the guanosine triphosphate (GTP)-bound active form and the guanosine diphosphate (GDP)-bound inactive form [20]. In its activated state (Rac1-GTP), this protein engages downstream effectors to orchestrate diverse cellular functions, including transcriptional regulation, cell cycle progression, and actin cytoskeleton-dependent cell migration [21]. Pharmacological inhibition of Rac1 signaling has emerged as a promising strategy for ameliorating AD-associated memory deficits [22]. Notably, Rac1 critically regulates astrocytic morphogenesis and functional dynamics [23], with preliminary evidence indicating its hyperactivation in AD model astrocytes. However, the spatiotemporal regulation of Rac1 in AD-associated astrocytes remains unexplored.

The Toll-like receptor 4 (TLR4)/Rac1/NOD-like receptor protein 3 (NLRP3) signaling pathway integrates multiple molecular components that drive neuro-inflammatory responses in AD [24]. In animal models of AD, microglia display sustained Rac1 and TLR4 signaling pathway activation and pathological elevation of key inflammatory mediators, including IL-1β, IL-6, the NLRP3 inflammasome, reactive oxygen species (ROS), and TNF-α. Targeted inhibition of Rac1 or TLR4 significantly attenuates cognitive impairment in amyloid precursor protein/presenilin 1 (APP/PS1) mice [25]. Despite this, the mechanistic contributions of Rac1 and its associated pathways in astrocyte-mediated neuro-inflammation remain poorly characterized.

Elucidating the regulatory mechanisms of astrocyte Rac1 and the TLR4/Rac1/NLRP3 axis in AD pathophysiology will provide critical insights into disease pathogenesis and advance the development of novel therapeutic interventions.

## 2. Astrocytes and AD

### 2.1 Physiological Functions and Pathological Alterations of Astrocytes

Since the late 19^th^ century, astrocytes have been classified into two major subtypes, protoplasmic and fibrous, which exhibit differences in cell morphology and anatomical location. Fibrous astrocytes are mostly distributed in the cortex of the brain and spinal cord. These astrocytes possess long, slender processes with relatively few branches, and their cytoplasm is rich in glial filaments; thus, they are also referred to as spider cells. Protoplasmic astrocytes, also known as mossy cells, are located mainly in gray matter, exhibiting short, thick processes with abundant branches and fewer cytoplasmic glial filaments [26-28]. Under physiological conditions, astrocytes play multiple important roles. For example, astrocytes secrete neurotrophic growth factors that promote the survival of neurons and oligodendrocytes, which generate myelin sheaths [29]. In the glycolytic pathway, astrocytes produce lactate and transport glucose, thereby providing energy support for neurons [30,31]. Moreover, astrocytes express glutamate transporters that recycle glutamate in the synaptic cleft [32] and directly regulate synaptic functions by releasing substances such as glutamate, γ-aminobutyric acid (GABA), and ATP. Astrocytes highly express inward-rectifying potassium channels that eliminate excess extracellular potassium ions, regulating the membrane potential and maintaining neuronal excitability [16]. Additionally, astrocytes can nourish neurons and regulate CBF by releasing vasodilators such as nitric oxide or arachidonic acid [33,34]. Nevertheless, astrocytes become reactive under pathological conditions. When affected by external stimuli, for example, pathological factors involved in AD-related damage, astrocytes often undergo activation reactions [35-37]. This is manifested in two main aspects. First, cell morphology changes and cytoplasmic fibrous substance accumulation occur, resulting in cell body hypertrophy, swelling, and an increase in the number and length of processes. Second, the expression of glial-related proteins, mainly glial fibrillary acidic protein (GFAP), vimentin, and S100β, is upregulated. The expression of these proteins in activated astrocytes is significantly greater than that in normal astrocytes, and these related proteins are often used to identify astrocytes in damaged areas of the central nervous system [38,39]. Furthermore, astrocytes play a protective role at sites of neuronal damage, where they proliferate to repair and isolate the damaged area, preventing it from spreading.

Recent studies have revealed that reactive astrocytes in AD can transform into disease-associated astrocytes (DAAs), primarily comprising two distinct subtypes: the proinflammatory A1 subtype (characterized by elevated expression of GFAP and complement component C3, which exacerbates neurotoxicity and synaptic loss) and the neuroprotective A2 subtype (marked by upregulated secretion of neurotrophic factors, including brain-derived neurotrophic factor (BDNF) and glial cell line-derived neurotrophic factor (GDNF), which facilitate tissue repair and anti-inflammatory responses) [40,41]. Single-cell sequencing analyses have revealed a unique population of *Gfap^+^ Apoe^+^* DAAs in the brains of AD patients, and the density of these cells is positively correlated with Aβ plaque burden [42]. In the context of AD pathology, Aβ deposition and chronic neuroinflammation synergistically drive the polarization of astrocytes toward the A1 phenotype, establishing their predominant distribution [40]. Paradoxically, A2 astrocytes may excessively produce GABA, which is released through Best1 channels, thereby inducing tonic synaptic inhibition and exacerbating cognitive deficits through dysregulation of neuronal network excitability [43,44]. In conclusion, astrocytes are dual-functional.

### 2.2 Astrocytic Clearance Mechanisms and Pathologic Transformation of Aβ Plaques in AD

Under physiological conditions, astrocytes clear Aβ plaques through multiple mechanisms [45]. Aβ clearance is mainly mediated by transporters, receptors, and the endosome–lysosome pathway, such as low-density lipoprotein receptor-related protein 1 (LRP1), class B scavenger receptor 1 (SCARB1), RAGE [46,47], neprilysin (NEP), insulin-degrading enzyme (IDE), and matrix metalloproteinase-9 (MMP9) [48,49]. Wyss-Coray et al. [50] placed astrocytes on APP transgenic mouse brain slices in an AD simulation experiment, which decreased the overall level of Aβ in the slices. Moreover, incubating adult astrocytes with Aβ1-42 *in vitro* decreased the level of Aβ1-42 within 3 hours, as demonstrated by western blotting (WB) and ELISA. These findings indicate that astrocytes are capable of degrading Aβ plaques deposited in vitro and in situ. However, under pathological conditions, astrocytes can contribute to Aβ deposition. Bruna Bellaver et al. [51] confirmed that the deposition of Aβ plaques is associated with increased astrocyte activity. The researchers divided participants into the astrocyte activity positive (Ast+) and negative (Ast-) groups for plasma Aβ42/40 and PET examinations, revealing that only Ast+ individuals demonstrated a significant increase in Aβ levels. Other related studies on cerebral cortex biopsy samples from AD patients have revealed that in the brain parenchyma and cerebral blood vessels, astrocytes adjacent to Aβ deposits exhibit ultrastructural alterations, such as atrophy, hyperplasia, and hypertrophy [52], as well as reduced expression of GLUT1 and lactate transporters [53,54]. These findings suggest that astrocyte dysfunction may be one of the causes of early behavioral and cognitive impairments in AD model mice.

Endothelin-converting enzymes (ECEs) are dual-role regulators of astrocyte-mediated Aβ metabolism, demonstrating spatiotemporal specificity. Previous studies have revealed upregulated ECE-2 mRNA and protein expression in neurons and reactive astrocytes within the temporal cortex of AD patients [55]. This molecular signature may be closely linked to Aβ oligomer-induced feedback regulation of protease activity. Notably, while it facilitates Aβ degradation, the ECE family also catalyzes the conversion of pro-endothelin to active endothelin-1 (ET-1). Recent evidence has revealed that the Rho GTPase signaling network mediates neurovascular unit dysfunction. Specifically, Aβ oligomers activate the Rac1/NADPH oxidase axis, triggering ROS overproduction and NF-κB inflammatory signaling cascades, thereby promoting aberrant ECE-1/2 expression and pathological ET-1 secretion [56]. This dysregulated signaling induces sustained cerebrovascular constriction, leading to significant reductions in CBF and impaired perivascular Aβ clearance efficiency. Consequently, a self-reinforcing (or vicious) cycle of “cerebrovascular constriction-cerebral hypoperfusion-exacerbated Aβ deposition” emerges. These findings collectively highlight that the dual regulatory mechanisms of ECEs, coupled with their associated Rho GTPase signaling network, explain the dynamic transition from protective compensation to pathological deterioration in AD pathogenesis. Spatiotemporally stratified intervention strategies—enhancing ECE-2 activity during the amyloid deposition phase and suppressing ET-1 secretion during the neurovascular injury phase—may represent novel therapeutic avenues to disrupt this pathological cascade

### 2.3 Astrocytes in Neuroinflammation and Synaptic Dysfunction

**Figure 1.**
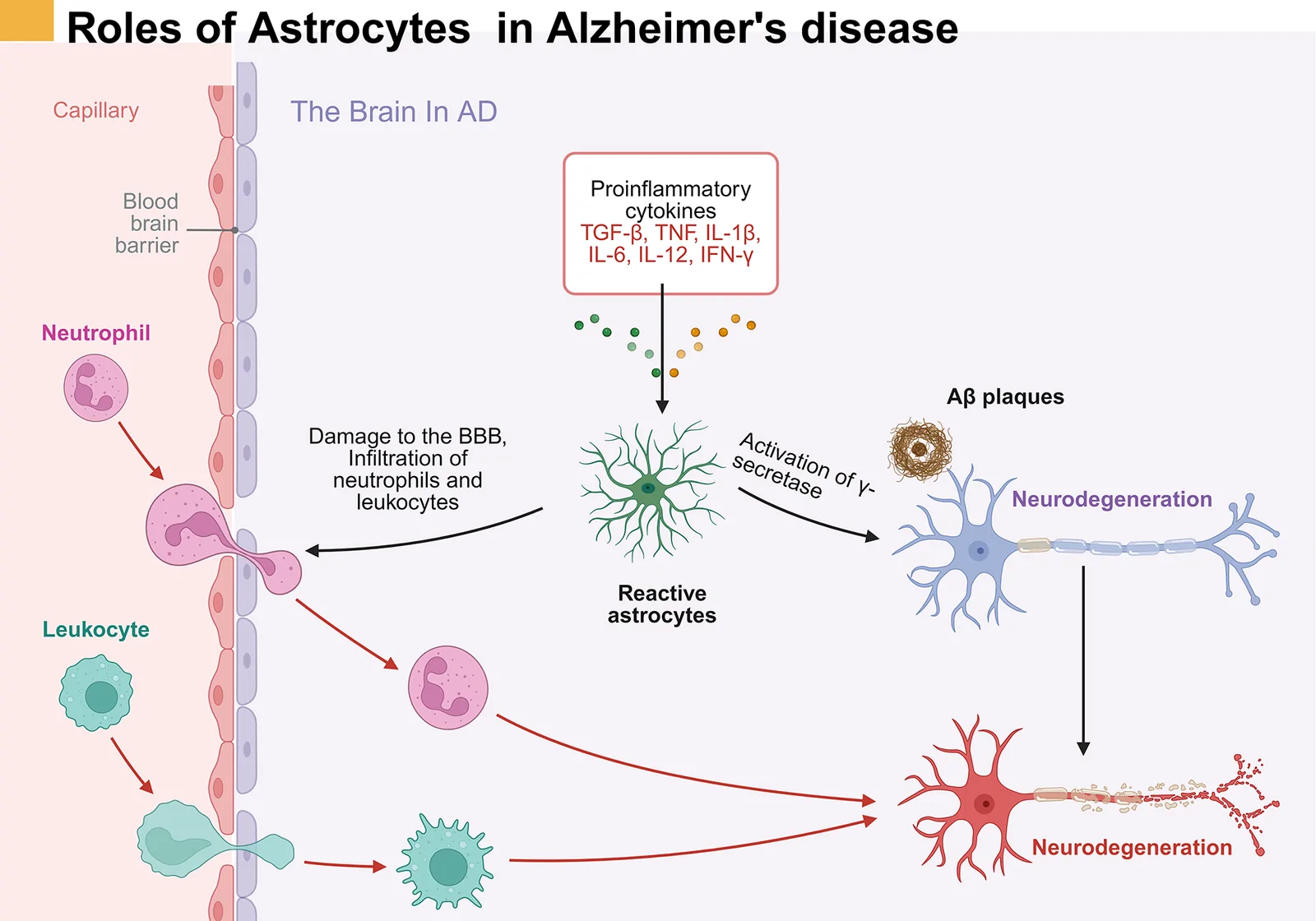
**Mechanisms of Astrocytes in AD Pathogenesis**. In AD, multiple proinflammatory cytokines, including TGF-β, TNF, IL-1β, IL-6, and IFN-γ, activate astrocytes, leading to the upregulation of IFITM3 expression and the activation of gamma-secretase. This cascade promotes Aβ accumulation, thereby inducing neuroinflammation and exacerbating AD pathogenesis. Concurrently, these processes contribute to BBB disruption and the infiltration of neutrophils and leukocytes. Created with BioRender.com.

In AD, various proinflammatory cytokines, including transforming growth factor-β (TGF-β), TNF, IL-1β, interleukin-6 (IL-6), and interferon-γ (IFN-γ), are capable of activating astrocytes [40]. This activation event increases the expression of interferon-induced transmembrane protein 3 (IFITM3), triggers the activation of γ-secretase, facilitates the accumulation of Aβ, and ultimately leads to neuroinflammation, thereby promoting the pathological progression of AD. Moreover, astrocytic activation can damage the BBB, accompanied by the infiltration of neutrophils and leukocytes (Fig. 1). Additionally, transcriptional analysis of reactive astrocytes isolated from AD model mice revealed that reactive astrocytes potently induce the expression of inflammatory genes such as Cst7, CCl4, Il1b, Clec7a, and Tyrobp, suggesting the involvement of astrocytes in the neuroinflammatory process of AD [57]. Furthermore, Aβ elicits astrocytic activation by binding to the TLR4 receptor, thereby driving its transformation toward a proinflammatory phenotype. Mechanistic studies have demonstrated that lipopolysaccharide (LPS), a TLR4 agonist, triggers TLR4/NF-κB pathway activation in astrocytes, accompanied by increased secretion of proinflammatory cytokines, including TNF-α and IL-6. Notably, these effects are abolished by anti-TLR4 antibodies, underscoring the pivotal role of TLR4 signaling in mediating Aβ-induced neuroinflammatory cascades [58,59]. These findings indicate that activated astrocytes can promote the formation of Aβ plaques and, in turn, that Aβ plaques can activate astrocytes [60,61]. This intricate interaction merits more in-depth exploration.

In AD animal models, reactive astrocytes release excessive GABA and glutamate, resulting in memory impairment and synaptic loss, thus exacerbating disease progression. As a specific chemically gated Ca^2+^ channel on the endoplasmic reticulum of cells, in astrocytes, calcium ion signaling mediated by inositol 1,4,5-trisphosphate receptor (IP3R) can accelerate the development of amyloid plaques and early synaptic plasticity deficits in APP/PS1 mice [62,63]. Downregulating IP3R in astrocytes can alleviate Aβ-induced calcium ion dysregulation and improve synaptic morphology and density.

## 3. Mechanism of Rac1 Protein functions in Astrocytes

### 3.1 Effects of Rac1 on the Morphology and Function of Astrocytes

#### 3.1.1 Rac1-Mediated Regulation of Astrocyte Morphology

**Figure 2.**
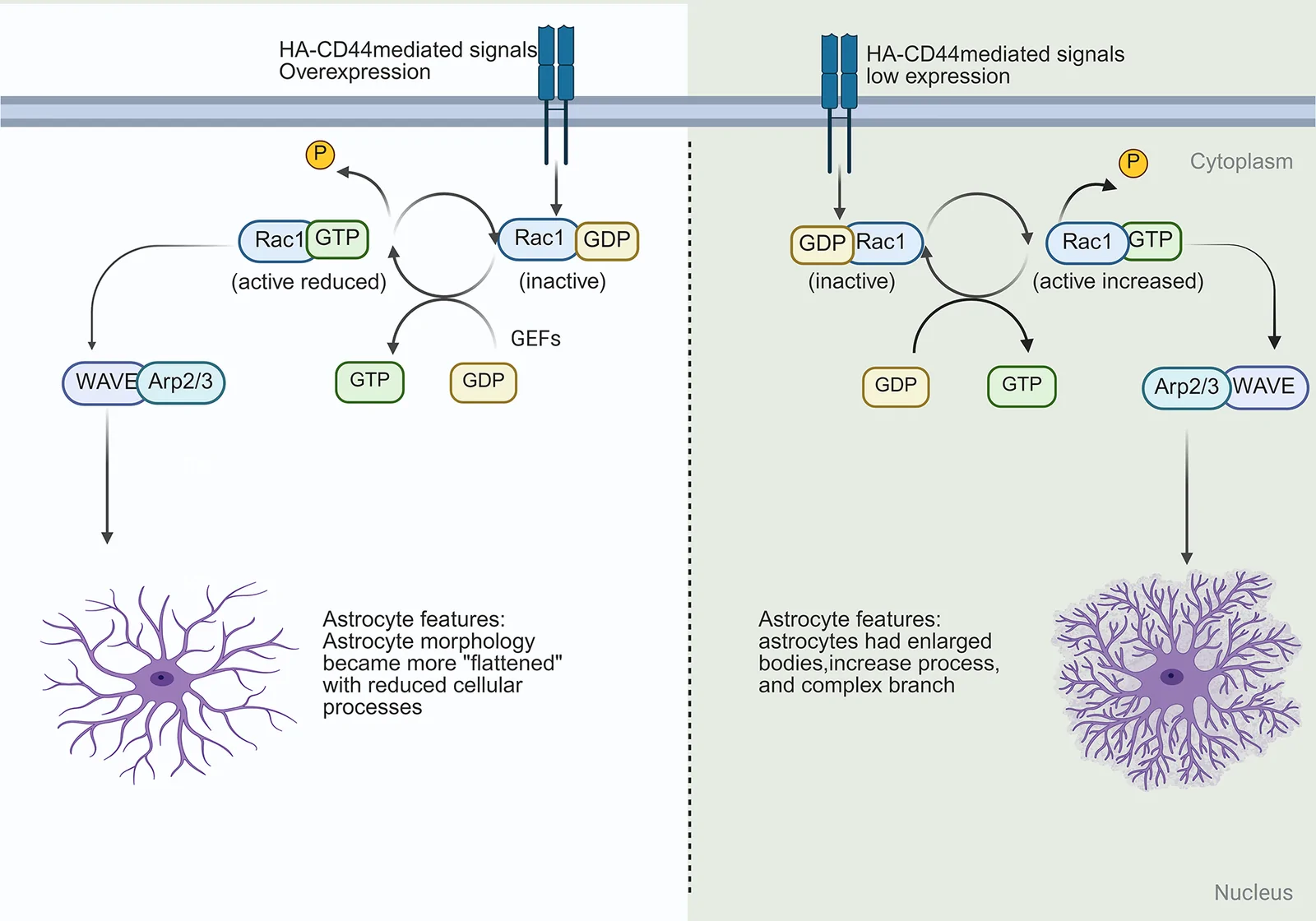
**Molecular Mechanisms of Rac1-Mediated Regulation of Astrocyte Morphology**. Rac1 regulates astrocyte morphology by modulating the dynamic reorganization of the actin cytoskeleton. In astrocytes, low expression levels of HA bound to surface CD44 receptors induce conformational changes in CD44. As a result, GEFs catalyze the GDP-to-GTP exchange on Rac1, transitioning Rac1 from a GDP-bound inactive state to a GTP-bound active state (enhanced activation). Rac1-GTP subsequently activates the WAVE complex (a member of the Wiskott-Aldrich syndrome protein family) and interacts with the Arp2/3 complex, thereby promoting de novo actin filament branching. These molecular events drive astrocytic morphological changes characterized by enlarged cell bodies, increased process complexity, and enhanced branching. Conversely, overexpression of HA-CD44 suppresses Rac1 activity, leading to a “flattened” astrocytic morphology with reduced process extension and branching complexity. Created in https://BioRender.com.

Rac1 influences the morphology of astrocytes by regulating the dynamic reorganization of the actin cytoskeleton [64,65]. CD44 expressed on the surface of astrocytes undergoes a conformational change upon binding to hyaluronic acid (HA). This change promotes the interaction between CD44 and Rac1-specific guanine nucleotide exchange factors (GEFs), such as Tiam1. Tiam1 then catalyzes the exchange of GDP for GTP on Rac1, converting Rac1 to its GTP-bound activated state [66]. Activated Rac1-GTP interacts with WAVE (a Wiskott-Aldrich syndrome protein family member) and the Arp2/3 complex [67,68], triggering the growth of new actin filament branches and ultimately altering the astrocytic cytoskeletal structure. Anna Konopka et al. [69] demonstrated that both hyaluronidase treatment and CD44 knockdown enhanced Rac1 activity, leading to an “astrocyte-like” morphology characterized by an enlarged cell body, increased processes, and complex branching. Conversely, overexpression of CD44 reduced Rac1 activity, and the cell morphology became more “flat” with fewer cell processes (Fig. 2). Xiao-Mu Guo et al. [70] reported that photoactivation of Rac1 could change the morphology of astrocytes, causing the cell membrane to extend outward. During contextual fear memory training, photoactivation of Rac1 in hippocampal astrocytes inhibited the formation of conditioned fear memory in mice. These findings suggest that the activity of Rac1 in hippocampal astrocytes may play a crucial role in regulating learning and memory processes.

#### 3.1.2 Rac1 Regulates Astrocytic Synaptic Homeostasis and Migratory Functions

Rac1 modulates posttraumatic hyperreflexia after spinal cord injury (SCI) by regulating astrocyte-mediated synaptic homeostasis. Astrocyte-specific Rac1 knockout (Rac1KO) in the ventral horn has been demonstrated to significantly upregulate the protein expression of the glutamate transporter GLT-1, thereby increasing glutamate clearance capacity and reducing extracellular glutamate concentrations at synaptic clefts. This effect suppresses the excessive activation of postsynaptic AMPA/NMDA receptors, ultimately alleviating the pathological hyperexcitability of α-motor neurons. Combined with Golgi staining for dendritic spine classification and spatial distribution analysis, these findings indicate that Rac1KO specifically reduces the SCI-induced aberrant proliferation of thin-type dendritic spines in the proximal and distal regions of α-motor neurons but has no significant effect on mushroom-type spine density. Electrophysiological recordings confirmed that this structural remodeling coincides with the restoration of frequency-dependent depression (FDD) of the H-reflex. Specifically, the Rac1KO group presented significantly lower H-wave amplitudes at 50 ms interstimulus intervals than the wild-type control group did, indicating marked mitigation of post-SCI hyperreflexia. These findings suggest that targeting the astrocytic Rac1 signaling pathway achieves precise intervention in pathological hyperreflexia through dual regulatory mechanisms, namely, GLT-1-mediated restoration of glutamatergic synaptic homeostasis and dendritic spine morphological remodeling, providing a spatiotemporally specific therapeutic target for clinical translation [71].

Further investigations revealed that Rac1 also coordinates astrocytic migration by integrating cytoskeletal remodeling and signal transduction. Celsr2KO significantly enhances the polarized migration of astrocytes via Rac1 pathway activation: in scratch assays, mutant cells presented 1.58-fold greater Rac1 activity (validated by WB), driving process elongation (104.1 vs. 78.2 μm) and a 44% increase in directional migration coverage. Pharmacological inhibition of Rac1 (NSC23766) restores the migratory capacity, process length, and directionality of Celsr2-/- astrocytes to wild-type levels [72]. These findings underscore the spatiotemporal-specific regulatory role of Rac1 in neural repair.

### 3.2 Rac1 Modulates Astrocyte-Neuron Metabolic Coupling

Astrocytes convert glucose into lactate via the glycolytic pathway, with lactate subsequently transported extracellularly through monocarboxylate transporters (MCTs), such as MCT1 and MCT4. Neurons then take up lactate via MCT2 for energy metabolism. This astrocyte-neuron lactate shuttle (ANLS) mechanism plays a pivotal role in supplying ATP substrates to neurons [73]. Rac1 orchestrates metabolic coupling by activating the downstream effector protein PAK1 and the WAVE complex, thereby driving dynamic remodeling of the microtubule/actin cytoskeleton. This process mediates the morphological transition of astrocytes from a flattened to a stellate phenotype. Concurrently, PI3 kinase cooperatively regulates regulatory exocytosis through enlargeosomes (regulatory secretory vesicles), facilitating the expansion of the plasma membrane surface area. Such morphological remodeling may modulate the transmembrane transport efficiency of lactate and other metabolites by altering the spatial distribution or abundance of membrane transporters (e.g., MCT1/MCT4) [74]. Notably, previous research has revealed reduced Rac1 activity in the cortical regions of postmortem AD human brains [75]. This suppression likely impairs cytoskeletal dynamics (via PAK1/WAVE complex inhibition) and disrupts membrane transporter localization (e.g., MCT1/MCT4), leading to glycolytic dysfunction and ANLS impairment, thereby exacerbating neuronal energy crises. Thus, restoring Rac1 activity or targeting its downstream effectors (e.g., PAK1, WAVE) may represent a novel therapeutic strategy for AD.

### 3.3 Molecular Mechanisms of Rac1-Mediated Astrocytic ATP Release via the PAK1/Cx43 Signaling Axis

Rac1 negatively regulates the opening of connexin 43 (Cx43) hemichannels on retinal astrocytes through its downstream effector PAK1, thereby suppressing the release of adenosine triphosphate (ATP) [76]. In a chronic ocular hypertension (COH)-induced glaucoma model, WB analysis revealed the biphasic downregulation of retinal Cx43 expression; there was an initial significant decrease at postoperative day 4 (G4d, *p*<0.01), followed by transient recovery to baseline levels at week 1 (G1w), and a then secondary decline at week 4 (G4w). Ethidium bromide uptake assays confirmed that abnormal phosphorylation at the Ser373 and Ser368 residues of Cx43 led to hemichannel dysfunction. Pharmacological inhibition or Rac1KO in astrocytes significantly increased Cx43 hemichannel opening and ATP release, which subsequently activated the adenosine A3 receptor on retinal ganglion cells (RGCs) and promoted their survival [76]. These findings indicate that astrocytes participate in regulating RGC injury during glaucoma pathogenesis through the Rac1/PAK1/Cx43/ATP signaling pathway. This discovery elucidates the critical role of glia–neuron interactions in glaucomatous neurodegeneration and provides a theoretical foundation for the development of novel neuroprotective strategies that target astrocytes.

### 3.4 Role of Rac1 in the Astrocyte-mediated Inflammatory Response

Rac1 plays a pivotal role in the astrocyte-mediated inflammatory response. Knockdown or inhibition of Rac1 can mitigate the inflammatory response in astrocytes. Wan et al. [77] first established a chronic inflammatory pain model induced by complete Freund's adjuvant (CFA). By intrathecally injecting an adenovirus interfering with Rac1 in astrocytes (AAV-GFAP-Mir30-Rac1) in rats, they discovered that knocking down Rac1 in spinal cord astrocytes could alleviate CFA-induced hyperalgesia. Moreover, Rac1 knockdown led to a decrease in the protein expression of GFAP and a decrease in the mRNA expression of the inflammatory factors TNF-α, IL-6, and IL-1β. Taiji Ishii et al. [78]reported that after LPS treatment, the expression of the proinflammatory factor IL-1β was reduced in the primary astrocytes of Rac1KO mice. These findings indicate that Rac1 is involved in the regulation of astrocyte-mediated inflammation.

#### 3.4.1 Molecular Mechanisms of Rac1-Driven Polarization Toward the A1 Proinflammatory Phenotype

Emerging evidence indicates that Rac1 acts as both a regulator of inflammatory cytokine expression and a critical driver of astrocytic polarization toward the A1 proinflammatory phenotype. For example, in chronic constriction injury (CCI) rat models and LPS-induced astrocyte activation assays, Rac1 hyperactivation has been found to significantly enhance the phosphorylation of the NF-κB p65 subunit, thereby promoting the transcription and secretion of proinflammatory cytokines, including IL-1β, TNF-α, and IL-6. These proinflammatory signals further induce astrocytic polarization into the A1 phenotype, characterized by marked upregulation of signature markers such as inducible nitric oxide synthase (iNOS), TLR4, and cyclooxygenase-2 (COX2), as demonstrated by significant increases in their protein expression levels in WB analyses [79]. Collectively, these findings provide critical molecular insights into targeting the Rac1/NF-κB pathway to modulate proinflammatory microenvironment remodeling in neurodegenerative diseases.

#### 3.4.2 β1 Integrin/PI3K/Rac1 Inflammatory Cascade: Molecular Pathway of Aβ Oligomer-Driven Astrocyte Activation

Emerging evidence reveals that, in addition to TLR4 signaling, the β1 integrin/PI3K/PKC axis represents another critical mechanism for Rac1 hyperactivation in astrocytes. Wyssenbach et al. [80] demonstrated in AD transgenic mouse models that Aβ specifically binds to β1 integrin receptors on astrocytic membranes, triggering a PI3K-dependent phosphorylation cascade of protein kinase C (PKC), which subsequently activates the Rac1 GTPase. Activated Rac1 drives the assembly of the NADPH oxidase (NOX2) complex, resulting in ROS overproduction and aberrant activation of the NF-κB signaling pathway. This activation ultimately induces the release of proinflammatory cytokines, including IL-1β and TNF-α. Targeting the β1 integrin/PI3K/PKC/Rac1/NOX axis may provide stage-specific therapeutic interventions for AD, such as blocking β1 integrin signaling in the early disease phases to mitigate the progression of oxidative stress.

## 4. The Role of the TLR4/Rac1/NLRP3 Pathway in AD

### 4.1 Overview of the TLR4/Rac1/NLRP3 Signaling Pathway

TLR4 is a pattern recognition receptor (PRR) predominantly expressed on the plasma membrane of hematopoietic cells, including macrophages, monocytes, and dendritic cells [81,82]. Its canonical ligand is LPS, a major component of the gram-negative bacterial cell wall [83,84]. Upon ligand binding, TLR4 initiates signaling cascades that activate nuclear factor-κB (NF-κB), driving the production of proinflammatory cytokines such as TNF-α, IL-1β, and IL-6 [81,85].

Rac1, a member of the Rho family of small GTPases, exists in two conformational states: an inactive GDP-bound state (Rac1-GDP) and an active GTP-bound state (Rac1-GTP) [86-88]. It regulates diverse cellular processes, including cytoskeletal remodeling, cell migration, and phagocytosis [89]. Among the TLR4-mediated signaling pathways, activated Rac1 modulates intracellular ROS generation [90]. ROS act as secondary messengers, facilitating signal transduction by directly inducing conformational changes in NLRP3 to promote inflammasome assembly. Additionally, ROS influence mitochondrial permeability transition pore (mPTP) opening, triggering the release of mitochondrial DNA (mtDNA) and mitochondrial ROS (mtROS) into the cytosol, both of which further activate the NLRP3 inflammasome [91].

**Figure 3.**
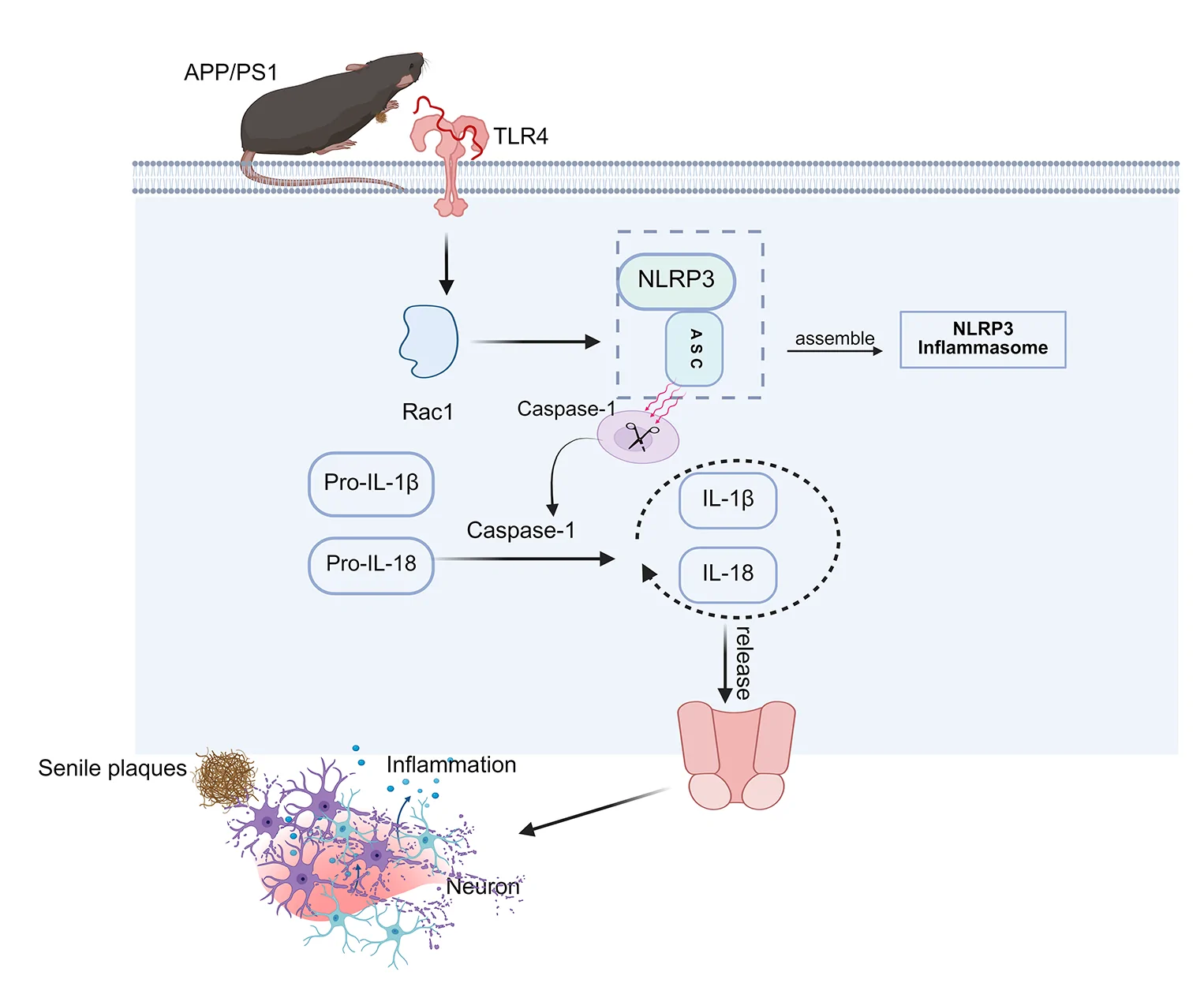
**Activation Mechanism of the TLR4/Rac1/NLRP3 Pathway in AD**. In AD, Aβ specifically binds to TLR4, inducing conformational changes in TLR4 that trigger a downstream signaling cascade. This cascade activates Rac1, which potentiates the activity of the NLRP3 inflammasome. The NLRP3 inflammasome recruits ASC and caspase-1 to form a functional complex. Activated caspase-1 then cleaves the precursor cytokines pro-IL-1β and pro-IL-18 into their mature forms, IL-1β and IL-18, driving neuroinflammation. Created with BioRender.com.

NLRP3, an intracellular PRR primarily localized in the cytoplasm of immune cells (e.g., macrophages and monocytes), is implicated in numerous human diseases [92]. Upon activation, NLRP3 oligomerizes with apoptosis-associated speck-like protein (ASC) and procaspase-1 to form the inflammasome complex, a critical step for NLRP3 functionality [93,94]. Activated caspase-1 subsequently cleaves pro-IL-1β into its mature, biologically active form, IL-1β, which is secreted extracellularly to amplify inflammatory responses (Fig. 3) [95].

### 4.2 Activation and Functional Implications of the TLR4/Rac1/NLRP3 Pathway in AD

#### 4.2.1 Activation Mechanisms

In AD, the deposition of Aβ aggregates serves as a pivotal pathogenic driver [96-99]. The NLRP3 inflammasome has been demonstrated to play a central role in AD progression [100,101]. In microglia specifically, Aβ binds to TLR4 with high specificity, inducing conformational changes in the receptor that initiate downstream signaling cascades. This activation promotes Rac1 hyperactivation, which in turn amplifies NLRP3 inflammasome activity. Subsequently, activated caspase-1 cleaves the proinflammatory cytokine precursors pro-IL-1β and pro-IL-18 into their mature, bioactive forms (IL-1β and IL-18), thereby driving neuroinflammatory responses (Figure 3) [25].

In astrocytes, activation of the TLR4/Rac1/NLRP3 pathway results in a unique indirect regulatory pattern: under AD pathological conditions, the TLR4 receptor preferentially initiates the MyD88-dependent early signaling pathway through its dual signaling architecture (comprising the MyD88-dependent early pathway and TRIF-dependent late pathway) [102]. Upon binding to the intracellular domain of TLR4 via its Toll/IL-1 receptor (TIR) homology region, MyD88 recruits IL-1 receptor-associated kinase (IRAK) family members [103], thereby initiating the assembly of the TRAF6/TAK1 signaling complex [84]. TAK1 subsequently drives NF-κB nuclear translocation, facilitating the transcriptional activation of proinflammatory cytokines [85], which exacerbates neuroinflammation. Notably, as a pivotal kinase downstream of the TLR4/MyD88 axis, TAK1 may indirectly increase Rac1-GEF activity through the activation of signaling nodes such as TRAF6 or NF-κB [104]. This interaction leads to sustained Rac1 hyperactivation, which promotes ROS generation and potentiates NLRP3 inflammasome assembly and activation [105]. Preclinical studies in AD models have demonstrated that pharmacological inhibition of TAK1 attenuates NLRP3-dependent IL-1β release and ameliorates cognitive deficits [106].

#### 4.2.2 Functional Impact of the TLR4/Rac1/NLRP3 Pathway in AD

##### 4.2.2.1 Neuroinflammation

Emerging evidence highlights the central role of the Aβ-TLR4 signaling axis in microglial activation and neuroinflammation. Dou et al. [107] demonstrated that Aβ_1-42_ activates the NLRP3 inflammasome via TLR4 binding, thereby promoting the release of proinflammatory cytokines (e.g., IL-1β and TNF-α) and inducing apoptosis. This process may involve TLR4-mediated regulation of cytoskeletal remodeling through downstream signaling pathways. Notably, Rac1 GTPase, a Rho family member, dynamically localizes to subcellular compartments (e.g., mitochondria, the plasma membrane, or perinuclear regions) through lipid modifications (e.g., geranylgeranylation) and protein interactions. These dynamics enable Rac1 to orchestrate oxidative stress responses, mitochondrial homeostasis, and inflammatory signaling [108]. Although no direct evidence links Aβ–TLR4 interactions to Rac1 spatial redistribution, it is plausible that Aβ_1-42_ alters Rac1 hyperactivation states or subcellular localization (e.g., cytosolic–mitochondrial translocation) via TLR4–dependent pathways (e.g., NF-κB or MAPK), thereby disrupting microglial cytoskeletal dynamics, phagocytic function, and inflammatory polarization. This hypothesis requires validation through subcellular colocalization assays (e.g., time-lapse imaging of Rac1 translocation from the cytosol to the mitochondrial membrane) or pharmacological Rac1 inhibition (e.g., NSC23766 treatment).

Notably, in this context, ponesimod, a sphingosine-1-phosphate receptor 1 (S1PR1) antagonist, exerts dual regulatory effects by targeting the S1PR1/TLR4 complex: it suppresses proinflammatory cytokine release by inhibiting the Stat1/p38 MAPK pathway while enhancing anti-inflammatory microglial polarization through Stat6 activation [109]. Such bidirectional modulation offers novel insights for precision therapeutic strategies in AD.

##### 4.2.2.2 Aβ Metabolism

Aberrant activation of the TLR4/Rac1/NLRP3 pathway exacerbates Aβ metabolic imbalance through two mechanisms: (1) Aβ deposition triggers microglial inflammatory responses via TLR4 signaling, forming a self-reinforcing “Aβ-TLR4-NLRP3” positive feedback loop that suppresses phagocytic function and disrupts autophagy–lysosome pathway (ALP) homeostasis, and (2) NLRP3 inflammasome activation releases proinflammatory cytokines (e.g., IL-1β), which further promotes Aβ generation [110]. A pioneering study by Freitag et al. demonstrated that the autophagy activator spermidine disrupts this vicious cycle by targeting microglia through the following mechanisms. (1) Inhibition of NLRP3 inflammasome assembly: spermidine induces selective autophagy to degrade ASC oligomers and suppresses TLR4/NF-κB signaling, thereby reducing the transcriptional synthesis of pro-IL-1β and procaspase-1. Concurrently, spermidine directly impedes ASC oligomerization, attenuating inflammasome activity and mature IL-1β/IL-18 release. (2) Enhanced Aβ clearance: single-nucleus RNA sequencing revealed that spermidine upregulates phagocytic receptors (e.g., AXL and TREM2) in disease-associated microglia (DAMs) while activating ARPC3-mediated actin cytoskeleton remodeling to promote microglial migration toward Aβ plaques. Proteomic profiling further confirmed that spermidine restores lysosomal function in microglia by modulating microtubule cytoskeleton organization and the ubiquitin–proteasome pathway, thereby accelerating Aβ degradation.

This study is the first to reveal that autophagy activation coordinately regulates inflammasome activity and phagocytic function to ameliorate Aβ metabolic dyshomeostasis, suggesting novel therapeutic approaches for AD by targeting the TLR4/Rac1/NLRP3 axis.

##### 4.2.2.3 Cognitive Function

Dysregulated activation of the TLR4/Rac1/NLRP3 pathway plays a pivotal role in cognitive impairment in AD. Evidence indicates that Aβ activates TLR4 signaling, further promoting Rac1 hyperactivation and enhancing NLRP3 inflammasome activity, thereby triggering neuroinflammatory cascades and neuronal damage [25]. In APP/PS1 model mice, pharmacological inhibition of TLR4 (CLI-095) or Rac1 (NSC23766) significantly reduces the expression of NLRP3 inflammasome-associated proteins (e.g., NLRP3, ASC, and caspase-1), decreases proinflammatory cytokine levels (IL-1β and IL-18), and improves spatial memory performance. Morris water maze assays demonstrated that pathway blockade shortened the escape latency while increasing the target quadrant dwell time and number of platform crossings, indicating cognitive recovery [25].

Notably, the cognitive benefits of *Tlr4*^-/-^ are corroborated in aging models. Fei et al. [111] reported that 16-month-old *Tlr4*^-/-^ mice exhibit superior learning and memory in Morris water maze tests, with elevated hippocampal synaptic density and upregulated memory-related proteins (PSD-95 and GluN2A). Furthermore, *Tlr4* deficiency enhances BBB integrity, reduces proinflammatory chemokine release (e.g., CXCL16 and CCL25), and improves CBF and synaptic plasticity, collectively mitigating age-related cognitive decline [111]. These findings collectively establish that the TLR4/Rac1/NLRP3 pathway exacerbates AD-associated cognitive deficits through neuroinflammation and synaptic injury, and its targeted inhibition represents a promising therapeutic strategy.

## 5. Challenges and Opportunities in Targeting the Rac1 or TLR4/Rac1/NLRP3 Axis in Clinical Settings: Perspectives on AD Heterogeneity and Patient Stratification

### 5.1 Challenges: AD Heterogeneity as a Bottleneck for Precision Intervention

The marked heterogeneity of AD, encompassing genetic background, pathological progression, and molecular phenotypes, poses a core challenge for targeted pathway interventions. For example, *in vitro* studies have demonstrated that microglia from *APOE4* carriers exhibit elevated TLR4 expression [112], leading to hyperactivation of the Rac1/NLRP3 pathway. Compared with noncarriers, these patients may show heightened sensitivity to TLR4 inhibitors [113,114]; however, they also face increased risks of drug toxicity. Additionally, AD patients exhibit a unique *GFAP^+^APOE^+^* astrocyte subtype [115], in which Rac1 activity is positively correlated with Aβ plaque burden [116]. Furthermore, regional heterogeneity in Rac1 signaling (e.g., cerebellum *vs*. cortex) [75] complicates the establishment of safe and effective therapeutic dose ranges.

The lag in patient stratification technologies further impedes clinical translation. Currently, reliable biomarkers (e.g., astrocyte-derived Rac1-GTP levels in cerebrospinal fluid) for distinguishing individuals with hyperactive TLR4/Rac1/NLRP3 pathways are lacking. The absence of such biomarkers hinders the precise identification of subtypes dominated by distinct genetic backgrounds or pathological mechanisms, resulting in nonselective therapeutic strategies. For example, while Rac1 inhibition improves cognitive function in APP/PS1 mouse models, it exacerbates metabolic dysfunction in astrocytes from *PSEN1*-mutant early-onset AD models. In these models, *TOMM40* and *PSEN1* cooperatively impair mitochondrial function, resulting in the disruption of energy metabolism [117]. Rac1 suppression may further compromise astrocytic glycogenolytic capacity, aggravating energy deficits [118]. Consequently, the same intervention paradoxically worsens astrocytic metabolic disturbances. Biomarkers such as Rac1-GTP could be used to predict whether patients belong to the Rac1-hyperactive subtype (characterized by elevated Rac1-GTP without *PSEN1* mutations), thereby avoiding therapeutic conflicts arising from genetic heterogeneity. Furthermore, the overlapping clinical phenotypes between AD and vascular dementia (e.g., cognitive decline and brain atrophy patterns) [119] pose diagnostic challenges. However, their inflammatory pathway activities differ markedly: AD is driven primarily by TLR4/NLRP3 activation [25], whereas vascular dementia involves ischemia-related inflammation [119]. Without specific biomarkers (e.g., NLRP3 oligomers or TLR4 phosphorylation status), anti-inflammatory therapies designed for AD may be misapplied to vascular dementia patients with ischemia-dominant pathology, leading to inefficacy or adverse outcomes.

### 5.2 Opportunities: Breakthroughs in Multidimensional Stratification Strategies and Precision Interventions

Single-cell RNA sequencing (scRNA-seq) has revolutionized research into AD heterogeneity. Recent studies utilizing scRNA-seq have identified two DAA subpopulations (A1 and A2 subtypes) in AD patient brain tissues. Among these subtypes, the A2 subtype results in upregulated complement C3 and downregulated synaptic-related gene *neurexin 1* (*NRXN1*), whereas the A1 subtype results in significant enrichment of the mitochondrial-related gene *MT-ND1-4* [120]. These discoveries provide a molecular foundation for precision subtyping: (1) The A2 subtype may serve as a biomarker for “synaptotoxic AD,” as its secreted complement component C3 and dysregulated *NRXN1* suggest synaptic impairment. Rac1, a key GTPase that regulates cytoskeletal remodeling, participates in astrocyte-mediated synaptic formation [121]. Studies have shown significantly elevated Rac1 activity in the hippocampi of AD patients [22], which correlates positively with complement C3 expression [122], suggesting their synergistic exacerbation of synaptic toxicity. (2) The A1 subtype may act as a biomarker for “energy metabolism-protective AD,” where upregulated mitochondrial genes such as *MT-ND1-4* likely reflect compensatory enhancement of mitochondrial function to counteract energy metabolism dysregulation or oxidative stress. Rac1, a critical component of the NADPH oxidase complex, promotes ROS generation upon activation [123]. Elevated ROS levels may adaptively upregulate *MT-ND1-4* to increase electron transport chain efficiency [124], maintaining the integrity of the mitochondrial network against oxidative stress. These findings indicate that Rac1 and *MT-ND1-4* cooperate to improve mitochondrial function, potentially delaying AD progression. Targeted interventions against the C3–Rac1 axis in the A2 subtype or enhancement of mitochondrial compensatory capacity in the A1 subtype could enable subtype-specific therapies, advancing the construction of a complete translational pipeline from molecular subtyping to clinical application.

## 6. Limitations of Current Research Models and Future Directions

### 6.1 Limitations of the Research Models

Current mechanistic investigations into the Rac1 signaling pathway predominantly rely on transgenic animal models (e.g., APP/PS1). However, these models demonstrate substantial discrepancies from human AD pathological hallmarks, thereby potentially diminishing the translational relevance of the derived conclusions. Specifically, APP/PS1 mice overexpress mutant APP and PS1 genes, which favor Aβ42 over Aβ40 production while lacking core AD neuropathological features such as tau hyperphosphorylation and neurofibrillary tangle formation. This limitation may result in an incomplete understanding of Rac1’s regulatory role in tau pathology. Furthermore, recent scRNA-seq has revealed A1/A2 functional subpopulations of astrocytes in AD patients, suggesting that Rac1 activity may modulate astrocytic subtype differentiation. Nevertheless, this mechanistic hypothesis remains unvalidated in animal models harboring human *APOE4* or tauopathy. Additionally, immortalized cell lines or primary isolated cultures of astrocytes fail to recapitulate the intricate neurovascular microenvironment *in vivo*, leading to oversimplification of the paracrine regulatory networks associated with the Rac1 signaling pathway.

### 6.2 Future Research Directions

To address the aforementioned limitations, the following development of research systems that better recapitulate human pathological features is urgently needed: (1) integration of spatial transcriptomics with multiphoton *in vivo* imaging to map spatial correlations between Rac1-hyperactive astrocytic subpopulations and Aβ/tau pathological deposits in postmortem AD brain sections; (2) generation of humanized cerebral organoids harboring *APOE4* to model gene–environment interactions regulating the Rac1 pathway in sporadic AD pathogenesis; and (3) the development of genetically encoded Rac1–GTP fluorescent biosensors for the real-time monitoring of spatiotemporal dynamics in astrocytic Rac1 activity within intact animal models via two-photon microscopy.

## 7. Conclusion

This review systematically elucidates the dual regulatory mechanisms of astrocytic Rac1 in the pathological progression of AD and its crosstalk with the TLR4/NLRP3 signaling pathway. Rac1, a key regulator of cytoskeletal remodeling, plays a pivotal role in AD pathogenesis by dynamically modulating astrocytic morphological plasticity (e.g., Rac1-GTP activation via CD44/HA-mediated processes leading to hyperplasia) and functional heterogeneity (e.g., calcium signaling dysregulation-induced synaptic dysfunction). Notably, the observed Rac1 hyperactivation in AD animal models may exacerbate disease progression through the following mechanisms: (1) Dysregulation of the oxidative stress–inflammatory axis: Aβ activates the Rac1/NADPH oxidase axis, triggering ROS overproduction and NF–κB inflammatory signaling cascades. This upregulation of astrocytic ECE-1/2 leads to aberrant ET-1 secretion, sustained cerebral vasoconstriction (reduced CBF), and impaired perivascular Aβ clearance. (2) Phagocytosis-neuroinflammation vicious cycle: the β1-integrin/PI3K pathway mediates Rac1/NOX2 activation, impairing astrocytic Aβ phagocytosis. Consequently, persistent TLR4/NLRP3 inflammasome activation establishes a self-reinforcing loop between amyloid deposition and neuroinflammation. (3) Synaptic homeostasis disruption: Rac1 dysregulation disrupts the glutamate/GABA metabolic balance, exacerbating glutamate excitotoxicity while attenuating inhibitory signaling. This culminates in dendritic spine loss and impaired synaptic plasticity.

The hierarchical activation of the TLR4/Rac1/NLRP3 signaling axis has been identified as a central mechanism linking Aβ pathology to neuroinflammation. The specific binding of Aβ to TLR4 activates Rac1 via the MyD88-dependent pathway, which subsequently triggers NLRP3 inflammasome assembly through mitochondrial ROS generation and K^+^ efflux, ultimately leading to caspase-1-dependent maturation and the release of IL-1β. Notably, therapeutic interventions targeting this pathway (e.g., the Rac1 inhibitor NSC23766 or the TLR4 antagonist TAK) have demonstrated multifaceted benefits in AD models, including a marked reduction in hippocampal inflammatory cytokine levels, increased Aβ clearance efficiency, and improved cognitive function, as evidenced by a shortened escape latency in the Morris water maze.

However, critical questions remain to be answered: (1) the spatiotemporal dynamics of Rac1 subcellular localization in astrocytes and its coupling to mitochondrial function require elucidation; (2) whether TLR4/Rac1 signaling modulates long-term inflammatory memory in astrocytes via epigenetic modifications (e.g., histone acetylation) remains unexplored; and (3) potential differences in pathway activation patterns across AD subtypes (early versus late onset) warrants investigation. Future studies should integrate cutting-edge technologies (e.g., single-cell sequencing and optogenetics) to dissect the molecular details of Rac1 signaling networks in astrocyte-specific spatiotemporal regulation, thereby advancing the development of cell type-selective precision therapeutics for AD.

## Data Availability

All the data generated or analyzed during this study are included in this published article [and its supplementary information files].
